# Presence-absence of marine macrozoobenthos does not generally predict abundance and biomass

**DOI:** 10.1038/s41598-018-21285-1

**Published:** 2018-02-14

**Authors:** Allert I. Bijleveld, Tanya J. Compton, Lise Klunder, Sander Holthuijsen, Job ten Horn, Anita Koolhaas, Anne Dekinga, Jaap van der Meer, Henk W. van der Veer

**Affiliations:** NIOZ Royal Netherlands Institute for Sea Research, Department of Coastal Systems, and Utrecht University, P.O. Box 59, 1790 AB Den Burg, The Netherlands

## Abstract

Many monitoring programmes of species abundance and biomass increasingly face financial pressures. Occupancy is often easier and cheaper to measure than abundance or biomass. We, therefore, explored whether measuring occupancy is a viable alternative to measuring abundance and biomass. Abundance- or biomass-occupancy relationships were studied for sixteen macrozoobenthos species collected across the entire Dutch Wadden Sea in eight consecutive summers. Because the form and strength of these relationships are scale-dependent, the analysis was completed at different spatiotemporal scales. Large differences in intercept and slope of abundance- or biomass-occupancy relationships were found. Abundance, not biomass, was generally positively correlated with occupancy. Only at the largest scale, seven species showed reasonably strong abundance-occupancy relationships with large coefficients of determination and small differences in observed and predicted values (RMSE). Otherwise, and at all the other scales, intraspecific abundance and biomass relationships were poor. Our results showed that there is no generic relationship between a species’ abundance or biomass and its occupancy. We discuss how ecological differences between species could cause such large variation in these relationships. Future technologies might allow estimating a species’ abundance or biomass directly from eDNA sampling data, but for now, we need to rely on traditional sampling technology.

## Introduction

Most conservation efforts depend on monitoring different species to obtain estimates of spatial distributions and population sizes^1^. Specimen collection and identification is expensive and labour intensive, and in practice monitoring programmes are constrained by the number of sampling units that can be afforded. As costs need to be reduced, many long-term monitoring programmes are now under pressure^2,3^. Cost-reductions could involve new technology that reduces the effort involved in species identification^1^, or shifting interest from estimating the abundance of animals to estimating their occupancy, i.e. the proportion of sampling sites in which a species was present^4^. Because measures of occupancy are often much easier and cheaper to measure than abundance or biomass^5^, the question arises whether occupancy is a reliable predictor of a species abundance or biomass, and whether occupancy sampling could thus reduce the cost of long-term monitoring programmes.

Occupancy-abundance relationships are among the most widespread empirical patterns described in macroecological studies^6–9^. The most commonly studied pattern is the interspecific (between species) abundance-occupancy relationship that describes how abundance and occupancy correlate between species in a particular area. Positive interspecific abundance-occupancy relationships are reported for many different taxa at different spatial scales and in a wide variety of ecosystems^8–12^. Even though the underlying mechanisms remain elusive^10^, possible ecological processes underlying positive abundance-occupancy patterns involve habitat use^6,13,14^ and population dynamics, e.g. colonization and extinction rates^8,10,15–17^.

Abundance-occupancy relationships have also been studied within species^18–21^. Such intraspecific relationships are divided into temporal and spatial relationships. The intraspecific temporal relationship describes the correlation between abundance and occupancy of a single species across time. Compared to between-species abundance-occupancy relationships, there have been fewer studies on intraspecific relationships. There is support for positive intraspecific relationships^18,22^, but the strength of these relationships depends on the characteristics of each species, e.g., life-history, dispersal, and longevity^9,12,23,24^. Negative relationships have, however, also been found^8,10^ and the generality of a positive relationship remains unresolved^25^. The intraspecific spatial relationship describes the correlation between abundance and occupancy of a species at a single point in time across space. Intraspecific spatial relationships are rarely studied and there is no agreement whether the shape of spatial intraspecific relationships should be positive^10^. Both types of intraspecific abundance-occupancy relationships have been studied mainly in terrestrial systems^26^, but rarely in marine systems^9,11,23,24^.

**Figure 1:**
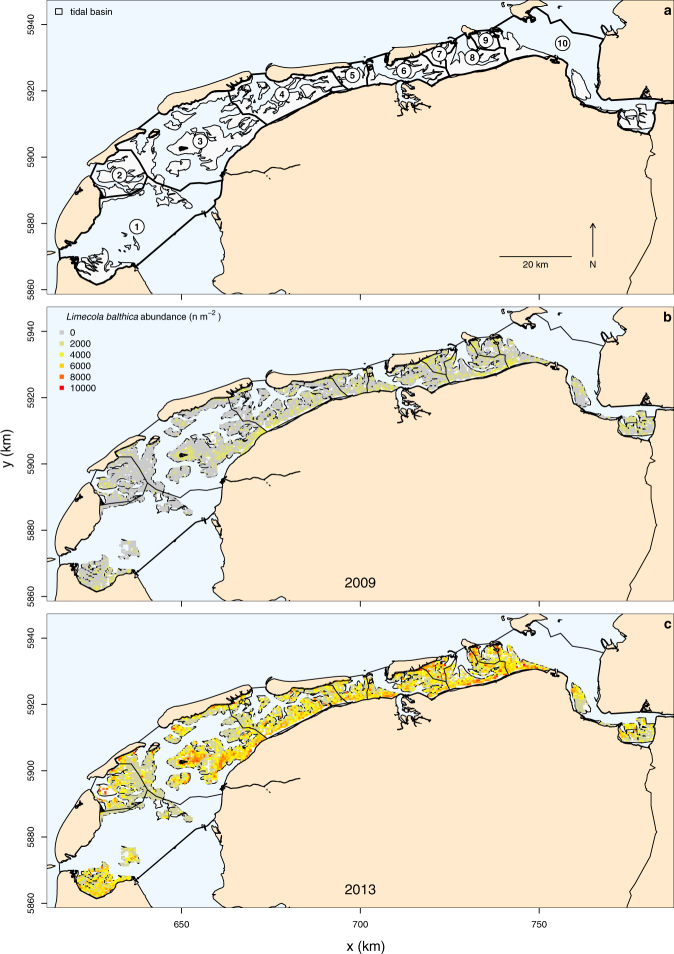
Study area with tidal basins and the **s**patial distribution of *Limecola balthica*. Panel a shows the study area and the various tidal basins: (1) Marsdiep, (2) Eierlandse Gat, (3) Vlie, (4) Borndiep, (5) Pinkegat, (6) Zoutkamperlaag, (7) Eilanderbalg, (8) Lauwers, (9) Schild, and (10) Eems-Dollard. Tidal mudflats are presented in light grey, permanently submerged areas in light blue, and exposed land in light brown. To illustrate spatial variation in abundance and occupancy, panel b shows the distribution of *L. balthica* abundance in 2009 (m^−2^). To show the simultaneous increase in abundance and occupancy for *L. balthica*, panel c presents its abundance for 2013. Each square represents a sampling location and its colour the abundance. The borders of the 10 tidal basins are superimposed. The maps were generated with R v3.2.3^60^.

In this study, we explore whether occupancy can provide accurate estimates of a species’ abundance and thus provide a cost-effective alternative to traditional sampling methods. We analysed intraspecific abundance-occupancy relationships^8^, also called distribution-abundance relationships^10^, for sixteen marine macrozoobenthos species that were collected across the Dutch Wadden Sea over a period of eight years^27,28^: the bivalves *Cerastoderma edule*, *Limecola balthica*, *Mya arenaria*, *Abra tenuis, Ensis leei, Mytilus edulis, Scrobicularia plana*, and *Macomangulus tenuis*, the polychaetes *Scoloplos armiger*, *Heteromastus filiformis*, *Hediste diversicolor*, *Nephtys hombergii*, *Lanice conchilega*, *Marenzelleria viridis*, and *Arenicola marina*, and the gastropod *Peringia ulvae*. Since the form and strength of abundance-occupancy relationships are scale-dependent^9^, they were analysed across different spatiotemporal scales. At the regional scale, yearly variation in abundance and occupancy was analysed across the entire Dutch Wadden Sea. At the local scale, yearly variation in abundance and occupancy was analysed within the ten tidal basins of the Dutch Wadden Sea (Fig. 1a). We also examined whether abundance-occupancy relationships occurred across geographic space at a single point in time. For this intraspecific spatial abundance-occupancy relationship, variation in abundance and occupancy between tidal basins within years was analysed. In abundance-occupancy modelling, abundance is generally measured as the density of the number of individuals^8^. However, many monitoring programmes are aimed at estimating a species’ biomass (e.g., as a possible food source for predators^29^), therefore biomass-occupancy relationships were also considered.

## Results

### Regional Temporal Relationships

**Figure 2:**
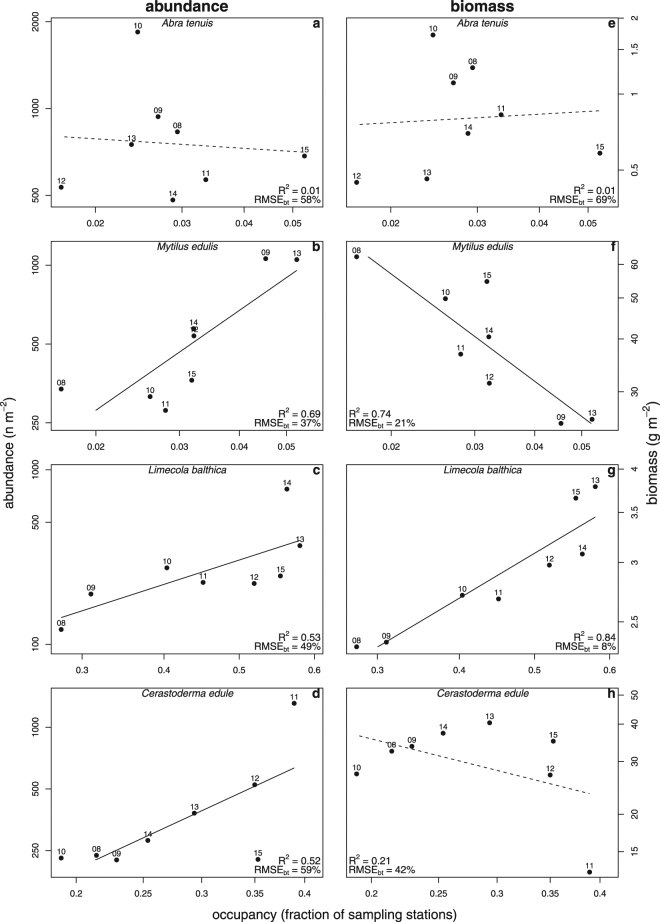
Regional intraspecific temporal relationships for a selection of four bivalve species (rows). Abundance-occupancy relationships are shown in the left column, and biomass-occupancy relationships in the right column. Each data point represents a yearly measurement of either a species’ abundance (m^−2^) or biomass (g m^−2^), and occupancy (fraction of sampling stations occupied) in the entire Dutch Wadden Sea. The log_10_ of abundance or biomass was modelled as a function of the logit of occupancy (solid line). To assess the strength of relationships, each panel shows the coefficient of determination (R^2^, proportion) and back-transformed Root Mean Squared Error (RMSE_bt_, %). Non-significance of linear models is indicated by dashed lines. Points are labelled with the last two digits of the sampling years.

**Figure 3:**
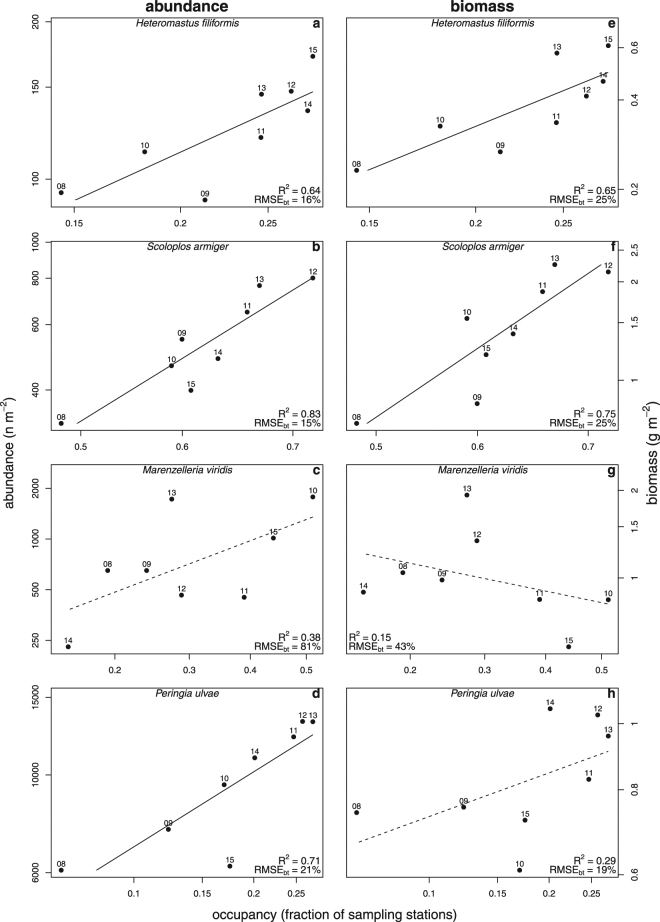
Regional intraspecific temporal relationships for three polychaetes and a gastropod (rows). Abundance-occupancy relationships are shown in the left column, and biomass-occupancy relationships in the right column. Each data point represents a yearly measurement of either abundance (m^−2^) or biomass (g m^−2^), and occupancy (fraction of sampling stations occupied) in the entire Dutch Wadden Sea. The log_10_ of abundance or biomass was modelled as a function of the logit of occupancy (solid line). To assess the strength of relationships, each panel shows the coefficient of determination (R^2^, proportion) and back-transformed Root Mean Squared Error (RMSE_bt_, %). Non-significance of the linear model is indicated by a dashed line. Points are labelled with the last two digits of the sampling years.

**Figure 4:**
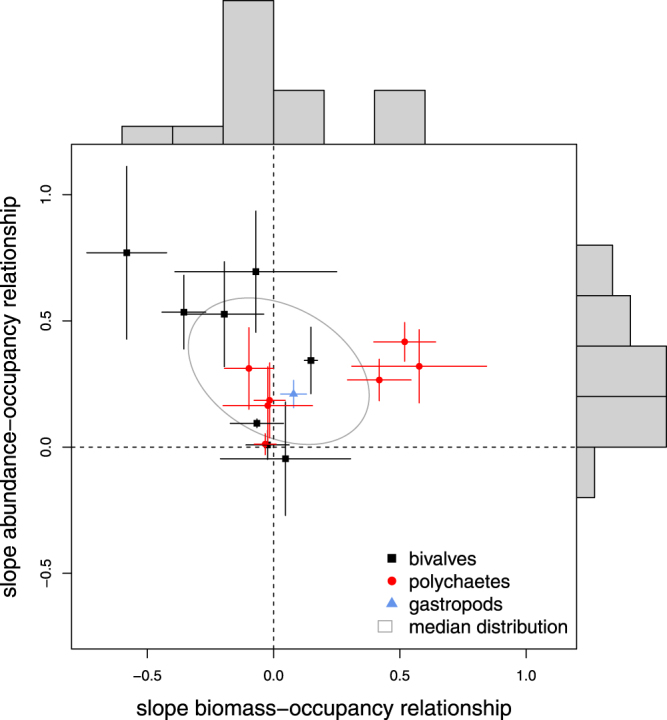
The association between the slopes of the intraspecific abundance-occupancy and biomass-occupancy relationships on a regional-scale. Each symbol represents a species as presented in Tables 1 and 2 with the estimated standard errors. Different plotting symbols represent different taxonomic groups (see legend). The ellipse describes the bivariate median distribution of slopes. Frequency distributions of the estimated slopes are presented on the right and upper axis for respectively the abundance-occupancy and biomass-occupancy relationships.

**Figure 5:**
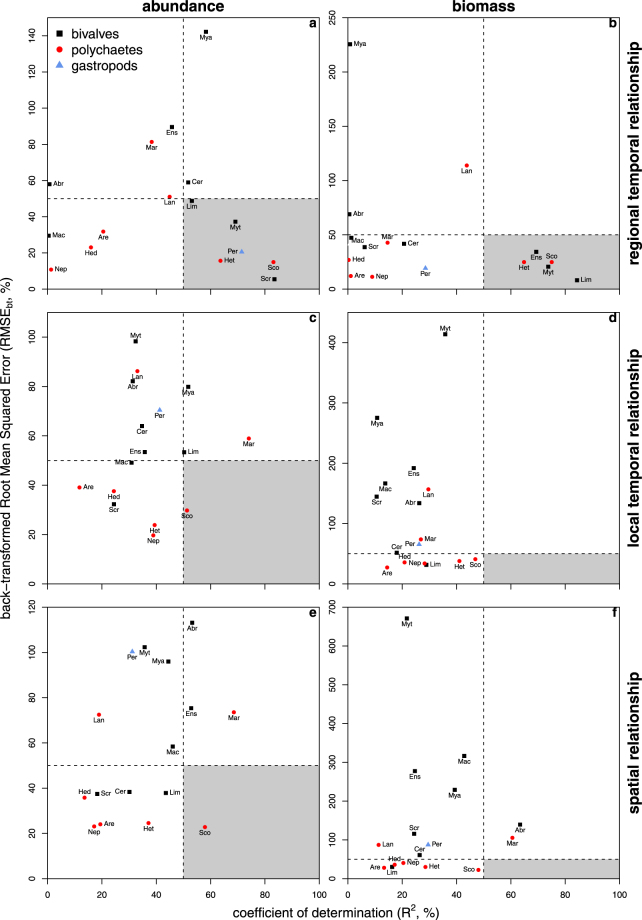
Abundance-occupancy (left column), and biomass-occupancy relationships (right column) for sixteen macrozoobenthic invertebrates. Upper panels show the temporal relationship on the scale of the entire Dutch Wadden Sea, middle panels the temporal relationship on the scale of tidal basins, and lower panels the spatial relationship across the Dutch Wadden Sea within years. Back-transformed Root Mean Squared Error (RMSE_bt_, %) are plotted against the coefficient of determination (R^2^, %). For the local temporal analyses and the spatial analyses, median values of RMSE_bt_ and R^2^ are plotted. To guide the eye, a horizontal and vertical line indicate the RMSE_bt_ and coefficient of determination of 50%. The quadrant with smallest RMSE_bt_ and highest R^2^ was shaded. Different plotting symbols represent different taxonomic groups (see legend).

On the regional scale of the entire Dutch Wadden Sea, the temporal relationships between abundance and occupancy were variable but mainly positive (Table 1, Figs 1b,c, 2a–d, 3a–d, 4, and Supplementary Figs. S1a–d and S2a–d). The steepest slopes were found for the bivalves *M. arenaria, M. edulis, C. edule*. and *E. leei*, and the polychaete *S. armiger* (Table 1). Strong relationships, characterised by a large coefficient of determination (>50%) and small back-transformed RMSE (<50%), were observed in the bivalves *M. edulis* and *S. plana*, the polychaetes *H. filiformis* and *S. armiger*, and the gastropod *P. ulvae*; Weak relationships, characterised by a small coefficient of determination and large RMSE_bt_, were observed in the bivalves *E. leei*, *M. arenaria* and *A. tenuis*, and the polychaete *M. viridis* (Table 1 and Fig. 5a). Interestingly, the coefficients of determination for the bivalves *M. arenaria*, *L. balthica* and *C. edule* were reasonably large (R^2^ > 0.5), but the RMSE_bt_ was also large (RMSE_bt_ > 49%, Table 1 and Fig. 5a). On average across the remaining species, small coefficients of variation and large RMSE_bt_ were found (Table 1 and Fig. 5a).

**Table 1:** Summary of mean yearly abundance and occupancy, and regional intraspecific temporal abundance-occupancy relationships (for the entire Dutch Wadden Sea). Yearly variance in abundance (log_10_ m^−2^) was modelled as a linear function of occupancy (logit of the fraction of occupied sampling sites). The estimated intercepts, slopes, coefficients of determination (R^2^, %), and the back-transformed Root Mean Squared Error (RMSE_bt_, %) are provided. Values in brackets are standard errors.

	Species	Abundance (m^−2^)	Occupancy (fraction)	Intercept	slope	R^2^ (%)	RMSE_bt_ (%)
Bivalves	*Abra tenuis*	24 (4)	0.03 (0.00)	2.72 (0.8)	−0.05 (0.22)	1	58
	*Cerastoderma edule*	138 (56)	0.28 (0.03)	3.04 (0.21)	0.53 (0.21)	52	59
	*Ensis leei*	39 (21)	0.12 (0.01)	3.88 (0.72)	0.77 (0.34)	46	90
	*Limecola balthica*	151 (45)	0.46 (0.04)	2.48 (0.07)	0.34 (0.13)	53	49
	*Macomangulus tenuis*	1 (0)	0.01 (0.00)	1.88 (0.28)	0.01 (0.06)	0	30
	*Mya arenaria*	114 (72)	0.11 (0.02)	4.08 (0.56)	0.69 (0.24)	58	142
	*Mytilus edulis*	22 ((7)	0.03 (0.00)	4.53 (0.5)	0.53 (0.15)	69	37
	*Scrobicularia plana*	2 (0)	0.03 (0.01)	2.15 (0.06)	0.09 (0.02)	84	5
Polychaetes	*Arenicola marina*	24 (3)	0.29 (0.02)	2.06 (0.14)	0.18 (0.15)	21	32
	*Hediste diversicolor*	76 (8)	0.34 (0.02)	2.44 ((0.11)	0.16 (0.15)	16	23
	*Heteromastus filiformis*	30 (4)	0.23 (0.02)	2.42 (0.10)	0.27 (0.08)	64	16
	*Lanice conchilega*	87 (20)	0.19 (0.02)	3.06 (0.23)	0.32 (0.14)	45	51
	*Marenzelleria viridis*	307 (103)	0.31 (0.04)	3.12 (0.17)	0.31 (0.16)	38	81
	*Nephtys hombergii*	7 (1)	0.12 (0.01)	1.77 (0.09)	0.01 (0.04)	1	11
	*Scoloplos armiger*	353 (50)	0.62 (0.02)	2.52 (0.04)	0.42 (0.08)	83	15
Gastropod	*Peringia ulvae*	2028 (429)	0.19 (0.03)	4.30 (0.09)	0.21 (0.05)	71	21

**Table 2:** Summary of mean biomass and regional intraspecific temporal biomass-occupancy relationships (for the entire Dutch Wadden Sea). Mean values for occupancy can be found in Table 1. Yearly variance in biomass (log_10_ g m^−2^) was modelled as a linear function of occupancy (logit of the fraction of occupied sampling sites). The estimated intercepts, slopes, coefficients of determination (R^2^, %), and the back-transformed Root Mean Squared Error (RMSE_bt_, %) are provided. Values in brackets are standard errors.

	Species	Biomass (g m^−2^)	intercept	slope	R^2^ (%)	RMSE_bt_ (%)
Bivalves	*Abra tenuis*	0.03 (0)	0.07 (0.92)	0.05 (0.26)	1	69
	*Cerastoderma edule*	8.51 (0.99)	1.28 (0.16)	−0.2 (0.16)	21	42
	*Ensis leei*	2.78 (0.3)	0.18 (0.33)	−0.58 (0.16)	69	34
	*Limecola balthica*	1.4 (0.2)	0.49 (0.01)	0.15 (0.03)	84	8
	*Macomangulus tenuis*	0.02 (0.01)	0.1 (0.42)	−0.02 (0.09)	1	47
	*Mya arenaria*	2.28 (1.25)	0.93 (0.74)	−0.07 (0.32)	1	226
	*Mytilus edulis*	1.24 (0.09)	0.37 (0.3)	−0.36 (0.09)	74	21
	*Scrobicularia plana*	0.28 (0.05)	0.68 (0.37)	−0.07 (0.11)	6	39
Polychaetes	*Arenicola marina*	2.5 (0.2)	0.91 (0.06)	−0.02 (0.06)	1	12
	*Hediste diversicolor*	1.01 (0.1)	0.44 (0.12)	−0.02 (0.18)	0	27
	*Heteromastus filiformis*	0.1 (0.02)	0.09 (0.16)	0.42 (0.13)	65	25
	*Lanice conchilega*	0.97 (0.33)	1.37 (0.42)	0.58 (0.27)	44	114
	*Marenzelleria viridis*	0.31 (0.05)	−0.09 (0.1)	−0.1 (0.1)	15	43
	*Nephtys hombergii*	0.18 (0.02)	0.09 (0.09)	−0.03 (0.04)	9	11
	*Scoloplos armiger*	0.96 (0.16)	−0.11 (0.07)	0.52 (0.12)	75	25
Gastropod	*Peringia ulvae*	0.16 (0.03)	0.04 (0.08)	0.08 (0.05)	29	19

At the regional scale, relationships between biomass and occupancy were highly variable and ranged from positive to negative (Table 2, Figs 2e–h, 3e–h, 4, and Supplementary Figs S1e–h and S2e–h). The steepest positive slopes were found for the polychaetes *L. conchilega* and *S. armiger*, and the steepest negative slopes were found for the bivalves *M. edulis* and *E. leei* (Table 2). Strong relationships were observed in the bivalves *L. balthica, M. edulis, E. leei*, and the polychaetes *H. filiformis* and *S. armiger*; Weak relationships were found in the bivalves *A. tenuis*, *M. arenaria*, and the polychaete *L. conchilega* (Table 2 and Fig. 5b). There were several species with small RMSE_bt_, but they also had small coefficients of determination (e.g., the polychaetes *A. marina*, *N. hombergii*, and the gastropod *P. ulvae*, Table 2 and Fig. 5b). For these species the overall mean was a better predictor of biomass than occupancy.

Comparing abundance-occupancy and biomass-occupancy relationships showed striking differences for some species (Fig. 4). Although for the bivalves *M. edulis*, *E. leei* and *C. edule* positive relationships were observed between abundance and occupancy, the relationships between biomass and occupancy were negative (Tables 1 and 2, Figs 2 and 4, and Supplementary Fig. S2). Compared to biomass-occupancy relationships, the RMSE_bt_ were similar but coefficients of determination were larger for abundance-occupancy relationships (median R^2^ of abundance and biomass relationships were respectively 0.49 and 0.18). The exceptions were the two bivalves *L. balthica* and *E. leii* that showed stronger occupancy-relationships for biomass than abundance (Tables 1 and 2, and Fig. 5a and b).

### Local Temporal Relationships

On the local scale within tidal basins, the between-year variation in abundance generally correlated positively with occupancy (Supplementary Fig. S3). However, between tidal basins and between species, we observed large variation in median intercepts (range = 1.85–4.29) and slopes (range = 0.04–0.40) (Supplementary Fig. S3). Additionally, the coefficients of determination were small (median R^2^ = 0.37) and Root Mean Squared Error large (median RMSE_bt_ = 51%, Fig. 5c). The weakest relationships were found for the bivalves *M. edulis* and *A. tenuis*, and the polychaete *L. conchilega*.

Compared to the regional scale, relationships between abundance and occupancy at local scales were more variable (the Inter Quartile Range of slopes were 0.30 and 0.12 for respectively local and regional relationships) as well as weaker (the median RMSE_bt_ of local and regional relationships were respectively 51 and 34%) with the exception of the bivalves *M. edulis, S. plana*, and the gastropod *P. ulvae* (Supplementary Fig. S5a).

For biomass, positive, negative or no relationships with occupancy were found within species across tidal basins (Supplementary Fig. S3). Between species, there was also a large variation in the relationships between biomass and occupancy (median slopes ranged from −0.18 to 0.40, Supplementary Fig. S3), and none of them were strong (Fig. 5d).

Compared to the regional scale relationships, relationships of between-year variation in biomass and occupancy within tidal basins were weaker with similar R^2^ but larger RMSE_bt_ (median RMSE_bt_ of the local and regional scale were respectively 63 and 31%, Supplementary Fig. S5b).

### Spatial Relationships

In general, positive spatial abundance-occupancy relationships were found, but these relationships were weak (median slopes ranged from −0.03 to 0.33, Supplementary Fig. S4) and highly variable between years. Only the polychaete *S. armiger* showed strong spatial abundance-occupancy relationships (R^2^ = 0.66 and RMSE_bt_ = 22%, Fig. 5e), but this was still weaker than its temporal relationship at the regional scale (R^2^ = 0.83 and RMSE_bt_ = 15%, Fig. 5a). Weakest relationships were found for the bivalves *M. edulis*, *M. arenaria*, the polychaete *L. conchilega*, and the gastropod *P. ulvae*.

Comparing spatial relationships with regional temporal relationships showed that spatial relationships were generally weaker, especially for the bivalves *M. edulis* (slope of respectively 0.29 and 0.53) and *S. plana* (slope of respectively 0.03 and 0.09), and the gastropod *P. ulvae* (slope of respectively 0.21 and 0.28) (Supplementary Fig. S5c). Only in the cases of the bivalve *E. leei* and the polychaete *M. viridis* did the R^2^ increase and RMSE_bt_ decrease, but these relationships were still weak (RMSE_bt_ > 68%, Fig. 5e).

The spatial relationships were similarly weak as the local temporal relationships (Supplementary Fig. S4c–f). Only for two polychaete species the spatial relationships were stronger than the local temporal relationships: *S. armiger* and *A. marina*, (Supplementary Fig. S5e). However, the regional temporal relationships for these two species were still stronger than both the spatial and local temporal (Fig. 5).

For biomass, the spatial biomass-occupancy relationships revealed large variation in median intercept (range = −0.28–2.57) and slope (range = −0.05–0.56, Supplementary Fig. S4). Moreover, none of the spatial biomass-occupancy relationships were strong (median R^2^ = 0.18 and RMSE_bt_ = 82%, Fig. 5f). One of the weakest relationships was observed for the bivalve *M. edulis* (R^2^ = 0.19 and RMSE_bt_ = 575%).

Compared to the regional temporal biomass-occupancy relationships, the spatial relationships were worse (RMSE_bt_ of respectively 31 and 82%), especially for the bivalves *M. edulis, E. leei* and *L. balthica* (Supplementary Fig. S5d). Also compared to the local temporal abundance-occupancy relationships (median RMSE_bt_ = 63%), the spatial relationships were weaker, e.g., for the bivalves *M. edulis* and *E. leei* (Supplementary Fig. S5f).

When comparing the spatial abundance-occupancy with spatial biomass-occupancy relationships, the biomass-occupancy relationships were more variable than the abundance-occupancy relationship. That is, the Inter Quartile Ranges of intercepts were 0.65 and 1.45 for the abundance and biomass relationships, 0.18 and 0.32 for the slopes, and 53% and 133% for the RMSE_bt_ respectively (Supplementary Fig. S4).

## Discussion

At the scale of the entire Dutch Wadden Sea, the intraspecific abundance-occupancy relationships were generally positive. Also, occupancy was usually positively related with biomass, but relationships were more variable than the abundance-occupancy relationships, and even negative for some species. The local temporal relationships and the spatial relationships were more often negative and weaker, in the cases of both abundance and biomass, than the regional temporal relationships. These findings suggest that occupancy data at large spatial scales could be informative about the abundance or biomass of selected species (e.g., the bivalves *L. balthica, S. plana*, *E. leei* and *M. edulis*, the polychaetes *S. armiger* and *H. filiformis*, and the gastropod *P. ulvae*.). However, for most species the predictive power of abundance and/or biomass from occupancy was low, i.e. the coefficients of determination were small and the difference in observed and predicted values large. Moreover, there were large differences in intercepts and slopes of the relationships between species. Within species, these relationships also varied between years and across geographic space, which further showed that there was a lack of generality for predicting a species’ abundance or biomass from its occupancy; especially at smaller scales.

Several modelling and empirical studies show that the slope of abundance-occupancy relationships is consistently shallower and weaker for rare species^12,20,30^. A related factor that could affect abundance-occupancy relationships is the distribution range (variation) of a species’ measured abundance and occupancy^8^. In our study, *A. tenuis* and *M. tenuis* were relatively rare with little variation in occupancy (respectively 2–5% and 0–3%), and indeed they showed shallow (slope close to zero) and weak relationships. However, even though *S. plana* was also rare and had a small occupancy range (2–5%), its abundance-occupancy relationship was among the strongest, but also with a shallow slope. Likewise, commonness and a large range of occupancies were no guarantee for strong abundance-occupancy and biomass-occupancy relationships. *M. viridis* had an occupancy range of 15–54% but weak abundance-occupancy and biomass-occupancy relationships.

The variation in abundance-occupancy patterns observed in this study could be understood by differences in life-histories between species. Theory predicts that abundance-occupancy relationships can be explained by: niche differentiation in resource and/or environmental use, which result in differences in vital rates and thus abundances along gradients of resources and/or the environment^6,8,10,13,15^, or population dynamics mediated by the movement of organisms between sites, which can be driven by competition for resources^14,17^. Based on the latter mechanism, weak abundance-occupancy relationships are predicted for species with low dispersal rates^16^. Thus a species that experiences little dispersion and aggregates locally is expected to have reduced occupancy compared to a more dispersive species^26^. A comparison between marine invertebrates showed that dispersal propensity affected abundance-occupancy relationships^23^. In our study, *A. tenuis* has limited dispersal capabilities (it deposits egg masses locally into the sediment^31^) and indeed showed a shallow slope and weak abundance-occupancy relationship. Likewise, the species that spawn in the water column and/or have a planktonic juvenile phase, in which the currents can disperse individuals over large distances^32–34^, had the strongest abundance-occupancy relationships (e.g., *L. balthica*, *M. edulis*, *P. ulvae*). As an example, *M. edulis* can have a planktonic larval phase of up to two months^35^ and can potentially disperse very far. Our findings are, however, not conclusive as some species (e.g., *M. tenuis, L. conchilega, M. viridis*) that have a planktonic phase and should be capable to disperse over large distances, showed weak abundance- or biomass-occupancy relationships.

For many macrozoobenthos species, recruitment dominates population dynamics^36,37^. Recruits are often superabundant and disproportionally affect abundance, biomass, and occupancy. That is, small but numerous recruits occupy large areas but with little contribution to biomass, and *vice versa* few adults contribute considerably to biomass but survive in restricted areas. Recruitment events could explain the opposing signs of abundance-occupancy versus biomass-occupancy relationships that were found for *M. edulis*, *E. leei* and *C. edule*. For instance, in 2011 *C. edule* had a uniquely strong recruitment with maximum densities of almost 19,000 juveniles per square meter^38^. Across the eight years of this study, 2011 had the largest abundance and occupancy, and indeed the smallest biomass as well. Similarly, old and large individuals can also dominate abundance, biomass and occupancy. Moreover, theory predicts that longevity would cause shallow slopes^12,16^. *M. arenaria* can live for 28 years, reach 15 cm in length^39^, and the abundance and occupancy of old individuals is small, but biomass is large. Indeed, the weakest relationships for *M. arenaria* was found. Moreover, longevity introduces strong temporal autocorrelation, due to cohort effects persisting through time, which might influence abundance-occupancy relationships further.

Local conditions can synchronize biomass-variation between macrozoobenthos species^40^, e.g., *C. edule, M. edulis, M. arenaria*. Therefore, local temporal relationships (within tidal basins) are predicted to be stronger than regional relationships (across the entire Dutch Wadden Sea), i.e. spatial variance is reduced. However, the abundance-occupancy relationships on the local scale were generally weaker, particularly for the above-mentioned species. Perhaps this is caused by smaller sample sizes as we scaled down to tidal basins. Alternatively, it could hint at large-scale processes that synchronise population dynamics of these species. For instance, the population dynamics of many marine macrozoobenthic species are affected by large-scale weather patterns. Cold winters can cause adult mortality, and mild winters can cause failed recruitment^40,41^. Large-scale weather patterns have indeed been found to synchronise population dynamics of different species over large spatial scales^40,42^, e.g., *M. edulis*, *M. arenaria*, *L. conchilega*, *C. edule*. Whether these large-scale processes underlie abundance-occupancy relationships, or perhaps influence their shape, needs to be investigated in further detail.

In this study, occupancy was measured in the traditional way by morphological taxonomy. Over the past years there has been an explosion in the use of environmental DNA (eDNA) metabarcoding as a tool for aiding monitoring programmes^1,43–48^. Studies have shown that eDNA sampling is accurate in collecting presence-absence data^49,50^, which could provide a cost-effective alternative to measuring occupancy. One should, however, be careful extrapolating our traditionally measured abundance-occupancy relationships to occupancies measured with eDNA techniques. There is some evidence that eDNA methods have higher detection rates than traditional field methods, particularly when species occur at low densities^51^. Sampling benthic invertebrates with small sediment cores can underestimate a species’ occupancy, i.e. imperfect detection leads to zero-inflated abundances^5,52^. This might especially be the case for *M. arenaria* that live partly below the reach of the sampling core (>30 cm) when they are very large (up to 15 cm)^39,53^. Indeed, this species showed the weakest abundance-occupancy relationships. The strength of abundance-occupancy relationships presented in this study could thus be underestimated compared to eDNA sampling techniques. Occupancy estimates could be improved by modelling detection probabilities^5^. To fully assess the validity of predicting abundance and biomass from eDNA occupancy data, traditional and eDNA sampling should be carried out simultaneously at the same locations. In the future, however, new genomic technologies could allow estimating abundance and biomass directly from eDNA sampling data^54,55^.

In summary, we find support for positive, as well as negative, intraspecific abundance- or biomass-occupancy relationships that could partly and non-conclusively be explained by ecological differences in life-histories between species. Abundance and biomass of some species could be accurately predicted from occupancy data, but only at the large scale of the entire Dutch Wadden Sea. At present, there is no generic relationship for predicting a species’ abundance or biomass from its occupancy. For the foreseeable, we therefore need to rely on traditional sampling technology for estimating a species’ abundance and/or biomass.

## Material and Methods

### Study System

The Dutch Wadden Sea (53°16′N, 5°24′E) covers roughly 2500 km^2^ of which 50% is tidal mud flats^56^. Due to natural tidal divides, the system is divided into ten physical units of tidal basins separated by watersheds (Fig. 1a).

### Field Sampling

From 2008 to 2015, the abundance and biomass of macrozoobenthic invertebrates were sampled across all 1151 km^2^ of intertidal mudflats in the Dutch Wadden Sea (Fig. 1) from June to September (SIBES Synoptic Intertidal BEnthic Survey)^27,28^. Sampling stations were arranged according to a grid sampling design with 0.5 km inter-sample distance and 15 to 20% additional sampling stations randomly placed onto gridlines^27^. In total between 3,159 and 4,818 stations were sampled and analysed for the sampling campaigns from 2008 to 2015, with the exception of 2015 where 1,289 samples of the random sample points have currently been analysed. In 2008, the Ems-Dollard estuary was not sampled. Within tidal basins, the surface area of the intertidal mudflats varies between 25 and 311 km^2^. Thus on average 75 to 1071 stations were sampled per tidal basin.

Sampling stations were located by handheld GPS (Garmin 60 and Dakota 10). At each station, two sediment cores (1/112 m² each) were taken to a depth of 25–30 cm, washed over a 1-mm square mesh sieve, and then transported to the laboratory. Because of the time between collection and processing, large bivalves were stored in the freezer and the polychaetes, crustaceans and small bivalves were kept on formalin.

In the laboratory, species were identified, individuals counted, and biomass (g) was determined as ash-free dry mass of the flesh (AFDM_flesh_)^28^. The shell and flesh of the gastropod *Peringia ulvae* were not separated, thus flesh was assumed to contribute 17% of the AFDM^57^. For bivalves, shell length (mm) was also measured.

### Analyses

Prior to analyses, outliers in AFDM_flesh_ were identified with a non-linear local regression of the log_10_ of AFDM_flesh_ and the log_10_ of shell length^58^ (R-script available in Supplementary Material Appendix A1). If residuals exceeded twice the Inter Quartile Range (IQR) they were defined as outliers. Because the lengths of polychaetes could not be accurately determined, they were divided into size-classes (juvenile or adult) and AFDM_flesh_ of outliers was estimated from their size class. *H. filiformis* could not be divided into size classes, therefore, outliers were estimated with mean log_10_ AFDM_flesh_. If AFDM_flesh_ was not measured, it was estimated from their length (bivalves) or size class (polychaetes). If both AFDM_flesh_ and length or size class were absent, the measurement was removed from the analyses (0.9% of measurements).

Abundance of a single species at a single sampling location was calculated as the number of individuals divided by the sampled surface area. To obtain the biomass of a single species at a single sampling location, the AFDM_flesh_ (g) of all individuals was summed. Mean abundance (n m^−2^) and biomass (g AFDM m^−2^) were then calculated by averaging abundances and biomasses of a species within occupied patches only (local mean abundance)^59^. To calculate presence-absence data, a species was classified as absent (0) or present (1) for each sampling station. Occupancy was calculated as the sum of the total number of presences divided by the number of stations visited.

For the analyses of intraspecific temporal relationships at the regional scale, mean abundance, biomass and occupancy of a species was calculated across the Dutch Wadden Sea in each year. To evaluate the local temporal relationship, abundances, biomasses and occupancy were averaged for each tidal basins in each year. The analyses of temporal relationships within tidal basins were restricted to those tidal basins where the species was observed at least six out of eight years. To examine spatial relationships, we examined the average abundance, biomass and occupancy between tidal basins within a single year, and then for each year separately to assess the yearly variation and robustness of these relationships across time.

Intraspecific relationships were modelled by fitting linear regressions between the log_10_ of abundance or biomass and the logit of occupancy. On the scale of tidal basins, abundance, biomass and occupancy data contained zeros. Before taking their logarithm or logit, we therefore added the smallest measured value of abundance or biomass within tidal basins, and for occupancy half times one over the sample size.

To evaluate the form of the abundance- or biomass-occupancy relationships, the intercept and slope were extracted from linear regression models. Because we were particularly interested in whether occupancy could predict abundance and/or biomass, we also extracted the coefficient of determination (R^2^) and the Root Mean Squared Error (RMSE). The coefficient of determination describes the proportion of variance in abundance or biomass explained by occupancy. For presentation purposes, the Root Mean Squared Error was back-transformed (RMSE_bt_) by taking the anti-log of RMSE, subtracted by 1, and multiplied by 100. The resulting RMSE_bt_ provided the percentage difference between observed and predicted values. A strong relationship should be characterised by a large R^2^ and small RMSE_bt_, whereas a weak relationship should be characterised by a small R^2^ and large RMSE_bt_.

All data was analysed in R v3.2.3^60^.

### Data availability

All data analysed in this study is available at 10.5281/zenodo.1120347.

## Electronic supplementary material

Supplementary Material
