# Supplementary material for: Presence-absence of marine macrozoobenthos does not generally predict abundance and biomass
Source: Sci Rep. 2018 Feb 14;8:3039. doi: 10.1038/s41598-018-21285-1 (PMC5813040; doi:10.1038/s41598-018-21285-1)
Supplement: Supplementary file 1 — Supplementary Material [file 41598_2018_21285_MOESM1_ESM.pdf]

SUPPLEMENTARY MATERIAL FOR:

Presence-absence of marine macrozoobenthos does not generally predict abundance and biomass

Allert I. Bijleveld*, Tanya J. Compton, Lise Klunder, Sander Holthuijsen, Job ten Horn, Anita Koolhaas, Anne Dekinga, Jaap van der Meer, and Henk W. van der Veer

NIOZ Royal Netherlands Institute for Sea Research, Department of Coastal Systems, and Utrecht University, P.O. Box 59, 1790 AB Den Burg, The Netherlands

Fig. S1 Regional intraspecific temporal relationships for bivalve species.
Fig. S2 Regional intraspecific temporal relationships for polychaete species.
Fig. S3 Local intraspecific temporal relationships.
Fig. S4 Spatial intraspecific relationships.
Fig. S5 The effect of scale on the strength of intraspecific relationships.
Appendix A1 R script for identifying outliers.

Fig. S1 Regional intraspecific temporal relationships for the bivalves species that were not plotted in Fig. 2. Abundance-occupancy relationships are shown in the left column, and biomass-occupancy relationships are shown in the right column. Each row represents one species. Each data point represents a yearly measurement of either a species' abundance (m^{-2}) or biomass (g m^{-2}), and occupancy (fraction of sampling stations occupied) in the entire Dutch Wadden Sea. We modelled the $\log_{10}$ of abundance or biomass as a function of the logit of occupancy (solid line). To assess the strength of relationships, each panel shows the coefficient of determination (R^2) and back-transformed Root Mean Squared Error ($RMSE_{bt}$, %). Non-significance of linear models is indicated by dashed lines. Points are labelled with the last two digits of the sampling years.

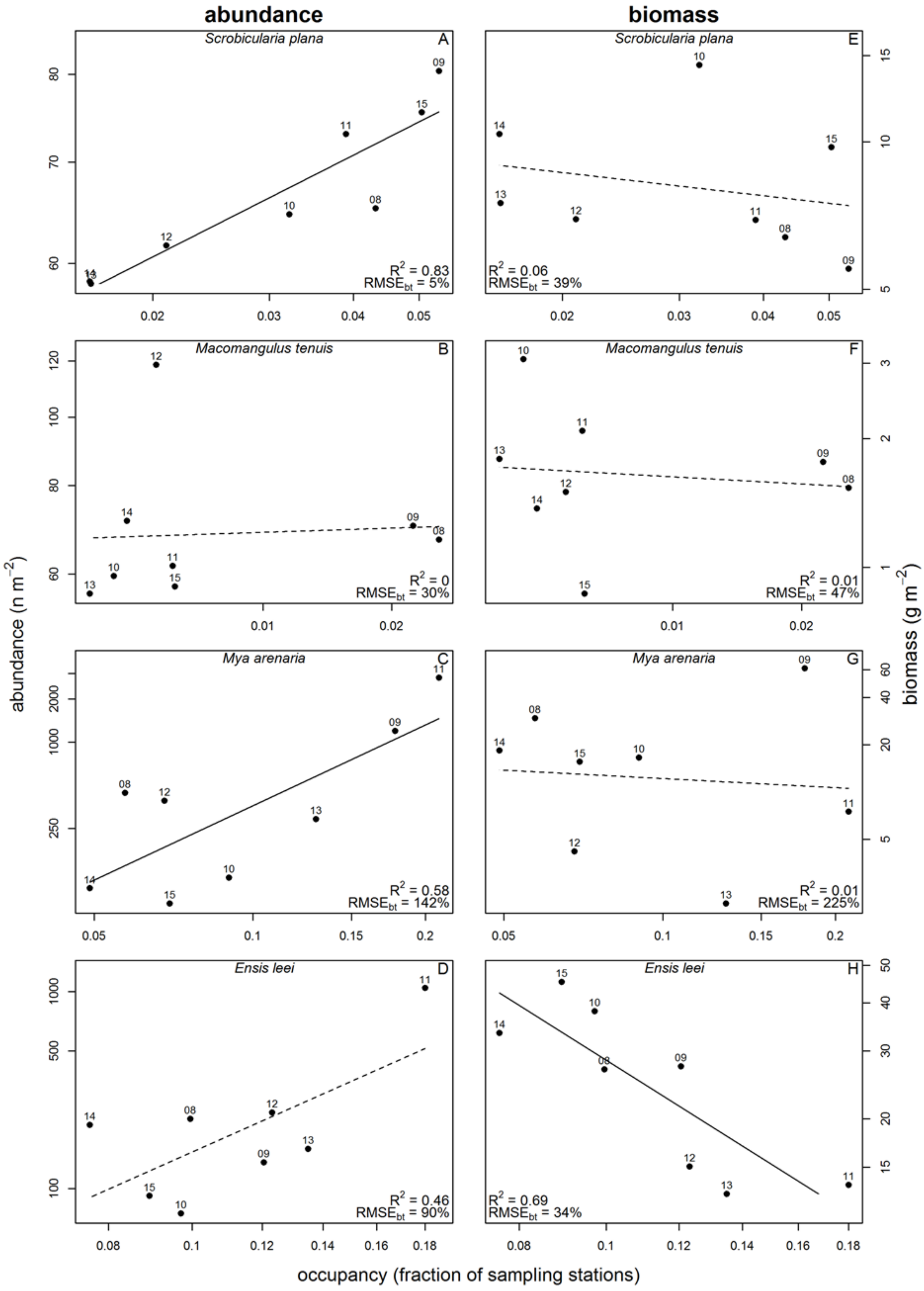

abundance
biomass
Scrobicularia plana
A
E
Macomangulus tenuis
B
F
Mya arenaria
C
G
Ensis leei
D
H
R² = 0.83
RMSEbt = 5%
R² = 0.06
RMSEbt = 39%
R² = 0
RMSEbt = 30%
R² = 0.01
RMSEbt = 47%
R² = 0.58
RMSEbt = 142%
R² = 0.01
RMSEbt = 225%
R² = 0.46
RMSEbt = 90%
R² = 0.69
RMSEbt = 34%
abundance (n m⁻²)
biomass (g m⁻²)
occupancy (fraction of sampling stations)

Fig. S2 Regional intraspecific temporal relationships for the polychaete species that were not plotted in Fig. 3. Abundance-occupancy relationships are shown in the left column, and biomass-occupancy relationships are shown in the right column. Each row represents one species. Each data point represents a yearly measurement of either a species' abundance (m^{-2}) or biomass ($g\ m^{-2}$), and occupancy (fraction of sampling stations occupied) in the entire Dutch Wadden Sea. We modelled the $\log_{10}$ of abundance or biomass as a function of the logit of occupancy (solid line). To assess the strength of relationships, each panel shows the coefficient of determination (R^2, proportion) and back-transformed Root Mean Squared Error ($RMSE_{bt}$, %). Non-significance of linear models is indicated by dashed lines. Points are labelled with the last two digits of the sampling years.

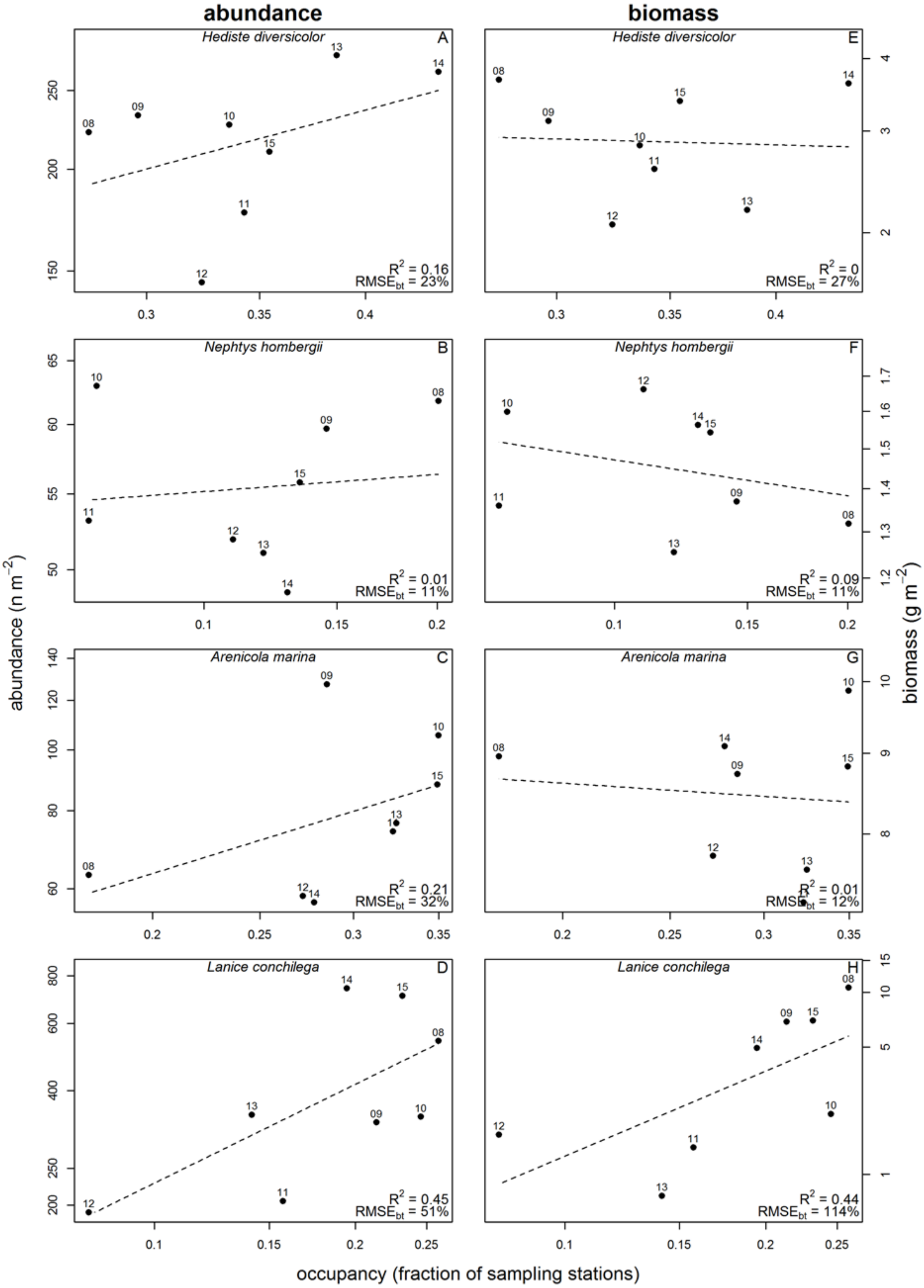
abundance
biomass
Hediste diversicolor
A
R^2 = 0.16
RMSE_bt = 23%
E
R^2 = 0
RMSE_bt = 27%
Nephtys hombergii
B
R^2 = 0.01
RMSE_bt = 11%
F
R^2 = 0.09
RMSE_bt = 11%
Arenicola marina
C
R^2 = 0.21
RMSE_bt = 32%
G
R^2 = 0.01
RMSE_bt = 12%
Lanice conchilega
D
R^2 = 0.45
RMSE_bt = 51%
H
R^2 = 0.44
RMSE_bt = 114%
abundance (n m^-2)
biomass (g m^-2)
occupancy (fraction of sampling stations)

Fig. S3 Local intraspecific temporal relationships. Abundance-occupancy relationships are shown in the left column, and biomass-occupancy relationships are shown in the right column. Each row of two panels represents one species. The lines represent tidal basins and are based on fitting linear regressions to eight data points of yearly measurements (2008-2015) of abundance (n m^{-2}) or biomass (g m^{-2}), and occupancy (fraction of sampling stations). To assess the variability and strength of relationships, each panel shows the mean coefficient of determination (R^2) and back-transformed Root Mean Squared Error ($RMSE_{bt}$, %) with their standard deviation between brackets. Tidal basins are numbered (as shown in Fig. 1A) from west (dark red) to east (blue). Note that abundance-occupancy relationships were not estimated if a species was not observed in more than two out of eight years (see methods).

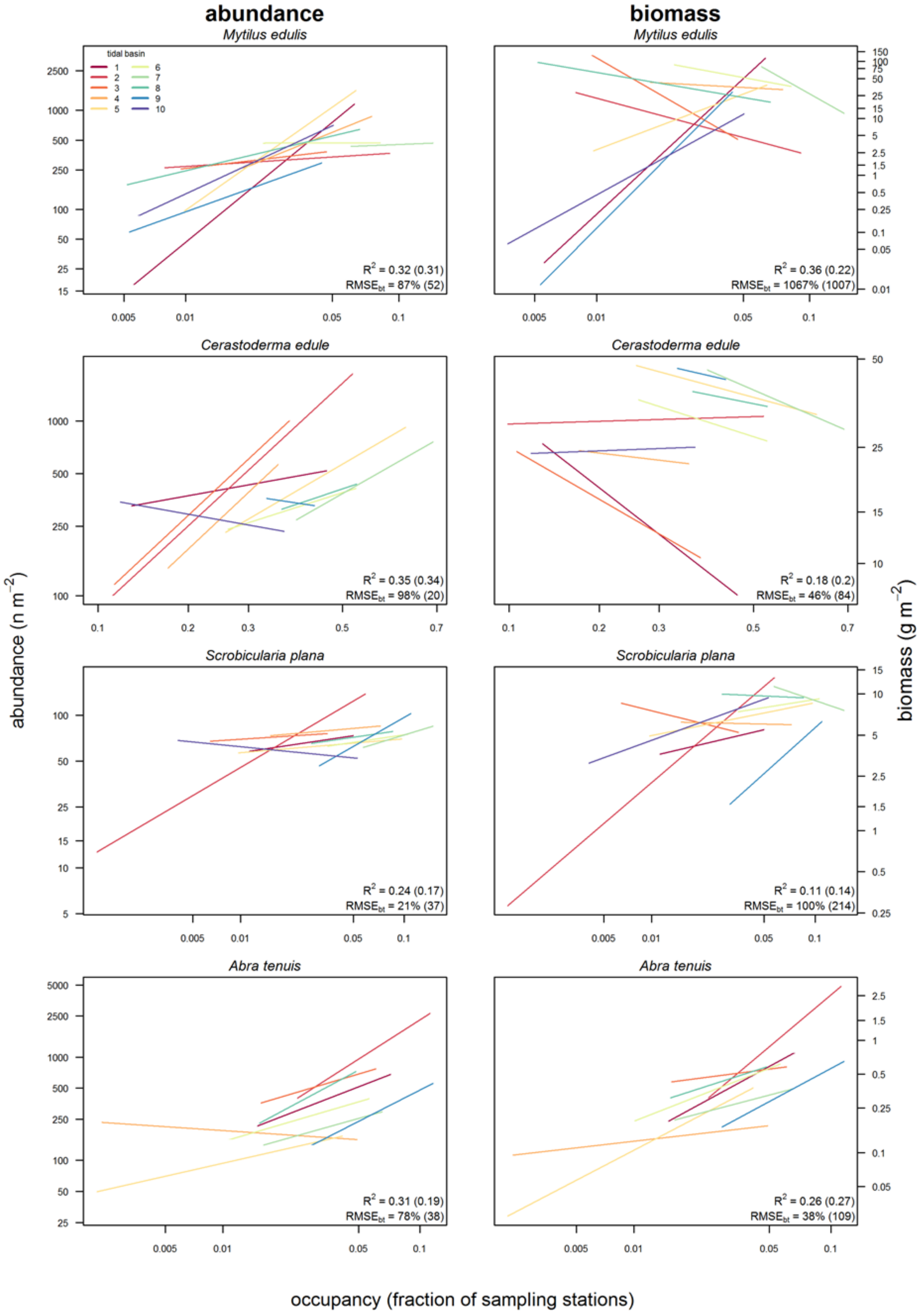
abundance
biomass
Mytilus edulis
tidal basin
1
2
3
4
5
6
7
8
9
10
R² = 0.32 (0.31)
RMSE_bt = 87% (52)
R² = 0.36 (0.22)
RMSE_bt = 1067% (1007)
Cerastoderma edule
R² = 0.35 (0.34)
RMSE_bt = 98% (20)
R² = 0.18 (0.2)
RMSE_bt = 46% (84)
Scrobicularia plana
R² = 0.24 (0.17)
RMSE_bt = 21% (37)
R² = 0.11 (0.14)
RMSE_bt = 100% (214)
Abra tenuis
R² = 0.31 (0.19)
RMSE_bt = 78% (38)
R² = 0.26 (0.27)
RMSE_bt = 38% (109)
abundance (n m⁻²)
biomass (g m⁻²)
occupancy (fraction of sampling stations)

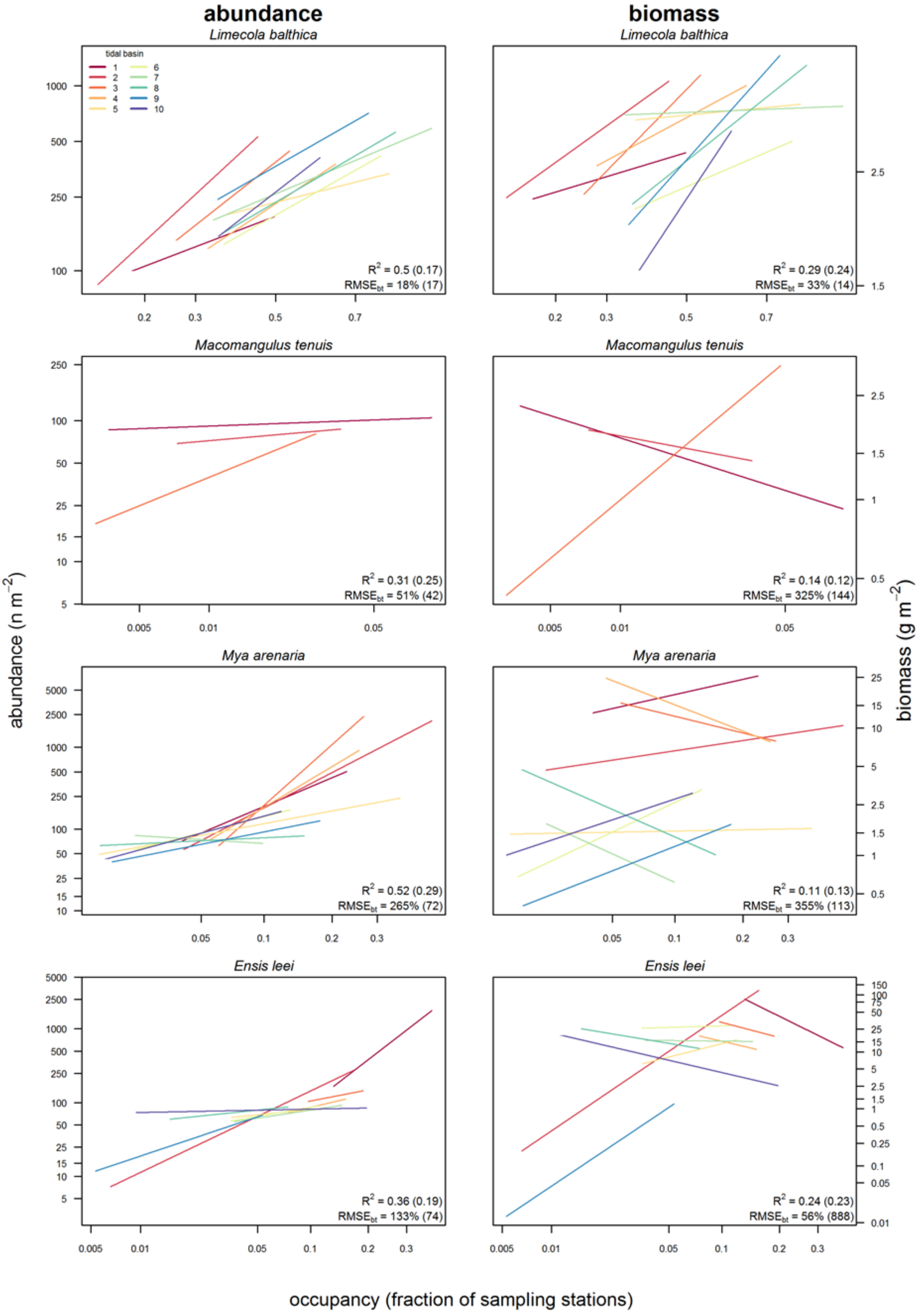
abundance
biomass
Limecola balthica
tidal basin
1
2
3
4
5
6
7
8
9
10
R² = 0.5 (0.17)
RMSEbt = 18% (17)
R² = 0.29 (0.24)
RMSEbt = 33% (14)
Macomangulus tenuis
R² = 0.31 (0.25)
RMSEbt = 51% (42)
R² = 0.14 (0.12)
RMSEbt = 325% (144)
Mya arenaria
R² = 0.52 (0.29)
RMSEbt = 265% (72)
R² = 0.11 (0.13)
RMSEbt = 355% (113)
Ensis leei
R² = 0.36 (0.19)
RMSEbt = 133% (74)
R² = 0.24 (0.23)
RMSEbt = 56% (888)
abundance (n m⁻²)
biomass (g m⁻²)
occupancy (fraction of sampling stations)

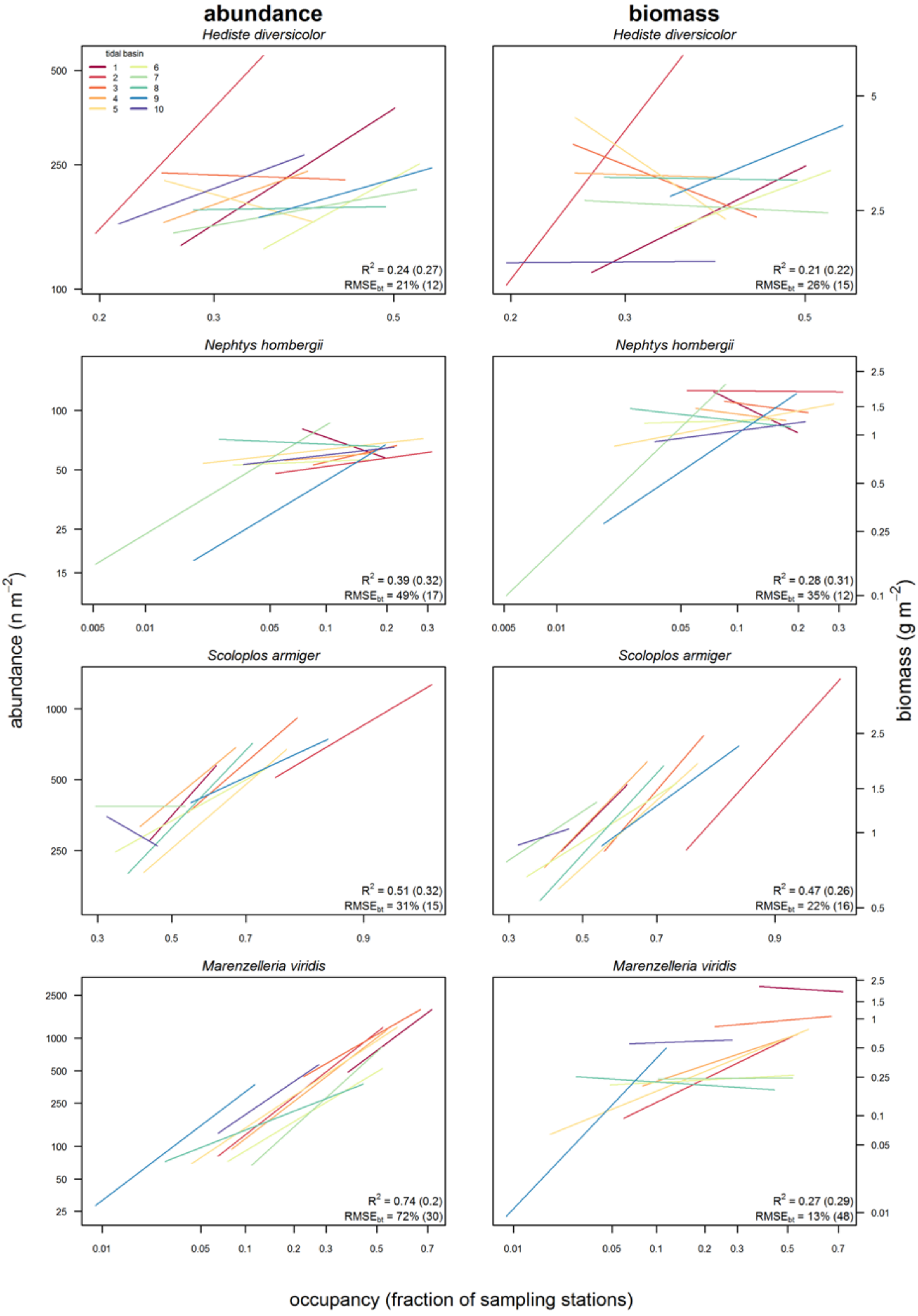
abundance
biomass
Hediste diversicolor
Hediste diversicolor
tidal basin
1
2
3
4
5
6
7
8
9
10
R² = 0.24 (0.27)
RMSEbt = 21% (12)
R² = 0.21 (0.22)
RMSEbt = 26% (15)
Nephtys hombergii
Nephtys hombergii
R² = 0.39 (0.32)
RMSEbt = 49% (17)
R² = 0.28 (0.31)
RMSEbt = 35% (12)
Scoloplos armiger
Scoloplos armiger
R² = 0.51 (0.32)
RMSEbt = 31% (15)
R² = 0.47 (0.26)
RMSEbt = 22% (16)
Marenzelleria viridis
Marenzelleria viridis
R² = 0.74 (0.2)
RMSEbt = 72% (30)
R² = 0.27 (0.29)
RMSEbt = 13% (48)
abundance (n m⁻²)
biomass (g m⁻²)
occupancy (fraction of sampling stations)

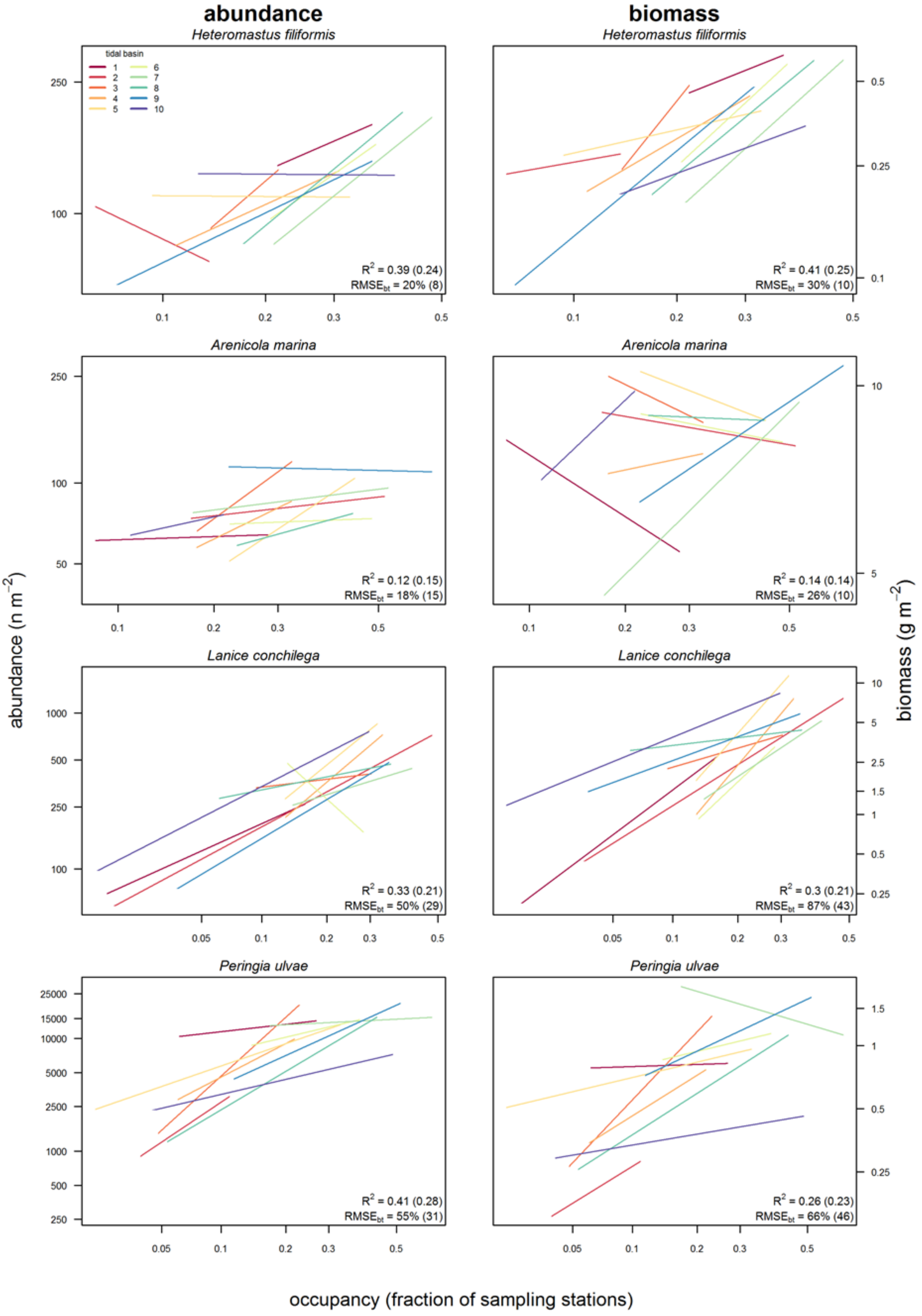

abundance
biomass
Heteromastus filiformis
Arenicola marina
Lanice conchilega
Peringia ulvae
tidal basin
1 2 3 4 5 6 7 8 9 10
R² = 0.39 (0.24)
RMSE_bt = 20% (8)
R² = 0.41 (0.25)
RMSE_bt = 30% (10)
R² = 0.12 (0.15)
RMSE_bt = 18% (15)
R² = 0.14 (0.14)
RMSE_bt = 26% (10)
R² = 0.33 (0.21)
RMSE_bt = 50% (29)
R² = 0.3 (0.21)
RMSE_bt = 87% (43)
R² = 0.41 (0.28)
RMSE_bt = 55% (31)
R² = 0.26 (0.23)
RMSE_bt = 66% (46)
abundance (n m⁻²)
biomass (g m⁻²)
occupancy (fraction of sampling stations)

Fig. S4 Spatial intraspecific relationships. Abundance-occupancy relationships are shown in the left column, and biomass-occupancy relationships are shown in the right column. Each row of two panels represents one species. The lines represent years and are based on fitting linear regressions to ten data points (one for each tidal basins, Fig 1A) of abundance (m^{-2}) or biomass ($g\ m^{-2}$), and occupancy (fraction of sampling stations occupied). To assess the variability and strength of relationships, each panel shows the average coefficient of determination (R^2, proportion) and back-transformed Root Mean Squared Error ($RMSE_{bt}$, %). The values in brackets represents standard deviations. Years are coloured from 2008 (dark red) to 2015 (blue).

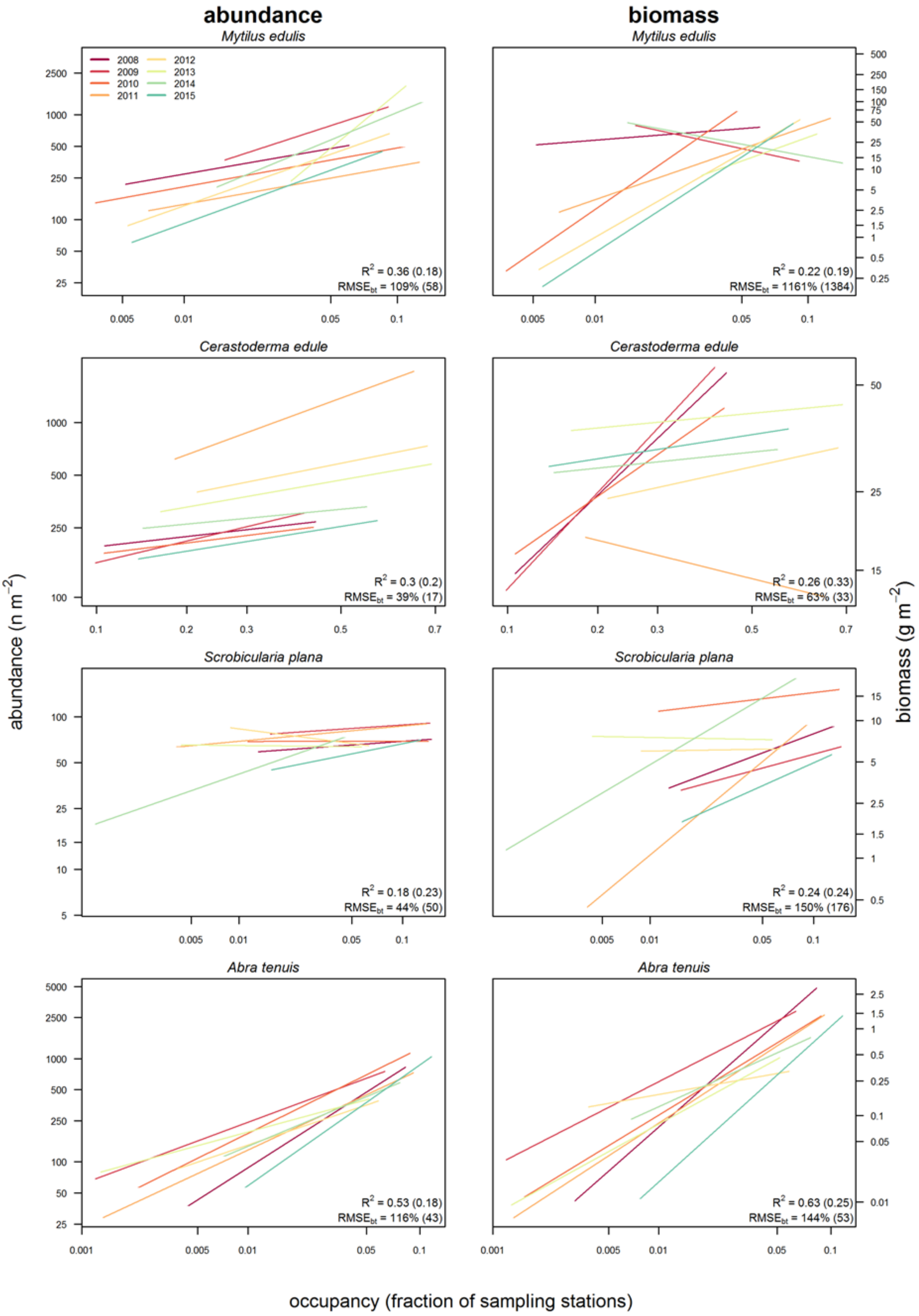
abundance
biomass
Mytilus edulis
Mytilus edulis
2008
2009
2010
2011
2012
2013
2014
2015
R² = 0.36 (0.18)
RMSEbt = 109% (58)
R² = 0.22 (0.19)
RMSEbt = 1161% (1384)
Cerastoderma edule
Cerastoderma edule
R² = 0.3 (0.2)
RMSEbt = 39% (17)
R² = 0.26 (0.33)
RMSEbt = 63% (33)
Scrobicularia plana
Scrobicularia plana
R² = 0.18 (0.23)
RMSEbt = 44% (50)
R² = 0.24 (0.24)
RMSEbt = 150% (176)
Abra tenuis
Abra tenuis
R² = 0.53 (0.18)
RMSEbt = 116% (43)
R² = 0.63 (0.25)
RMSEbt = 144% (53)
abundance (n m⁻²)
biomass (g m⁻²)
occupancy (fraction of sampling stations)

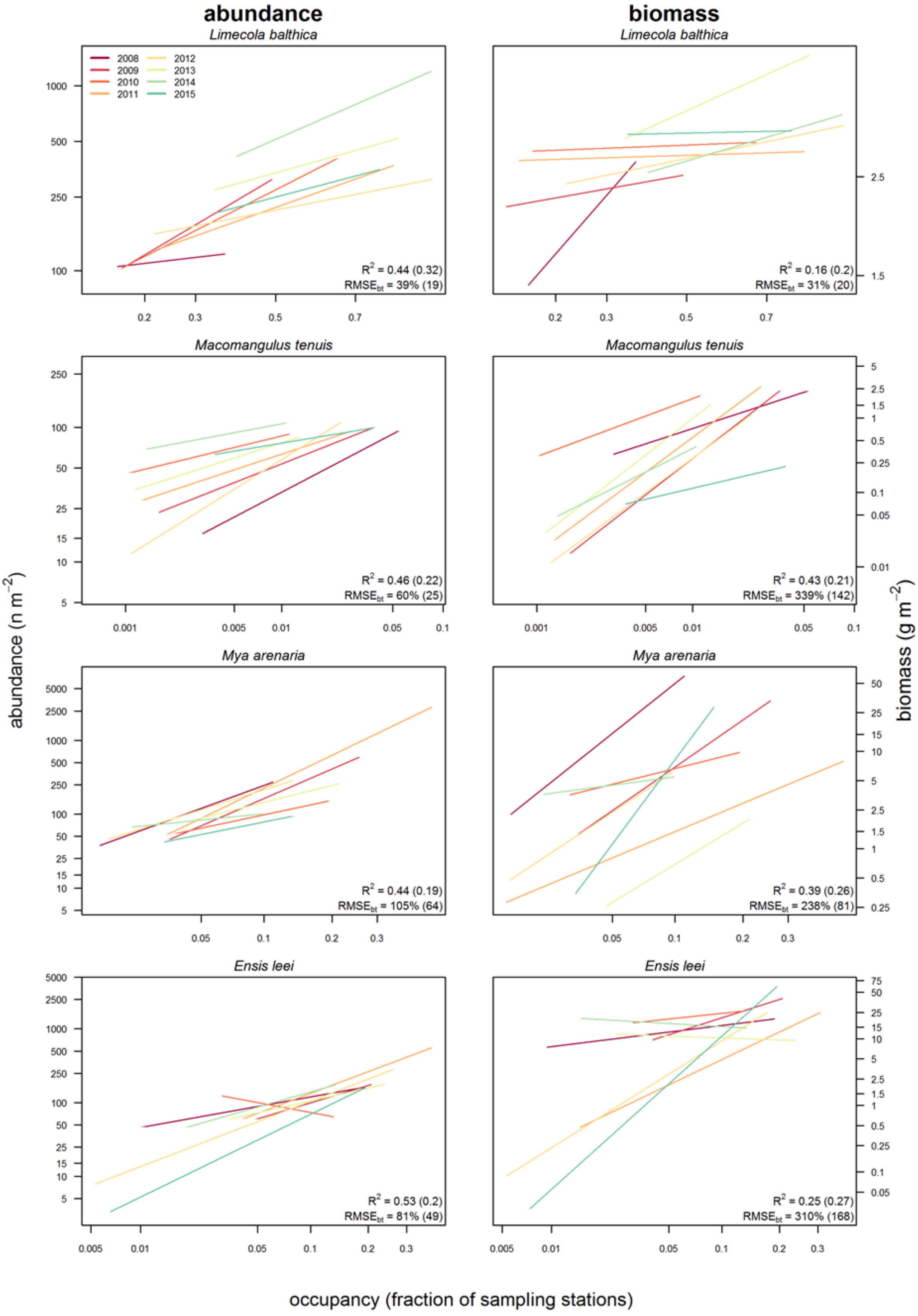

abundance
biomass
Limecola balthica
2008
2009
2010
2011
2012
2013
2014
2015
R² = 0.44 (0.32)
RMSEbt = 39% (19)
R² = 0.16 (0.2)
RMSEbt = 31% (20)
Macomangulus tenuis
R² = 0.46 (0.22)
RMSEbt = 60% (25)
R² = 0.43 (0.21)
RMSEbt = 339% (142)
Mya arenaria
R² = 0.44 (0.19)
RMSEbt = 105% (64)
R² = 0.39 (0.26)
RMSEbt = 238% (81)
Ensis leei
R² = 0.53 (0.2)
RMSEbt = 81% (49)
R² = 0.25 (0.27)
RMSEbt = 310% (168)
abundance (n m−2)
biomass (g m−2)
occupancy (fraction of sampling stations)

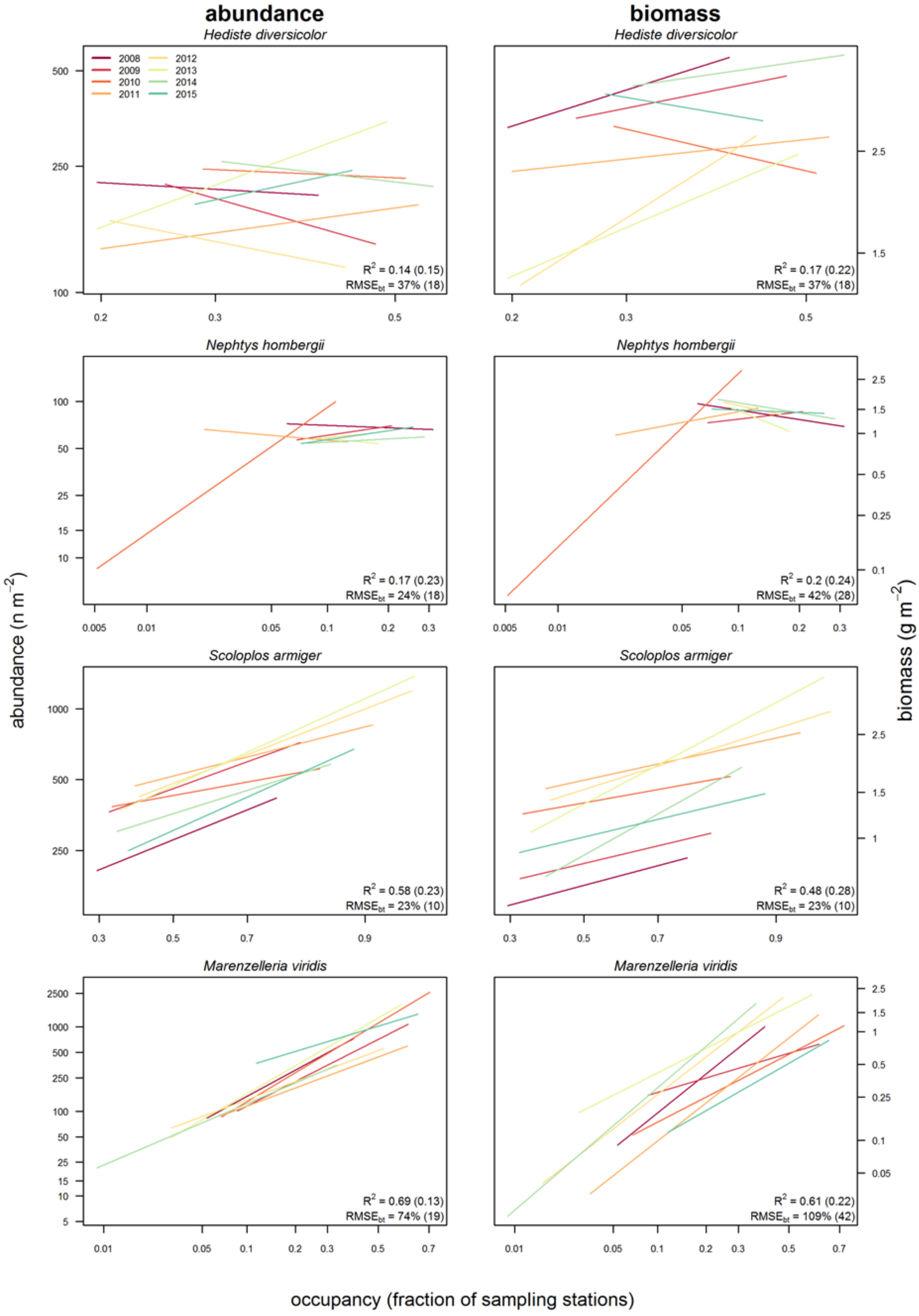

abundance
biomass
Hediste diversicolor
2008
2009
2010
2011
2012
2013
2014
2015
R^2 = 0.14 (0.15)
$RMSE_{bt}$ = 37% (18)
R^2 = 0.17 (0.22)
$RMSE_{bt}$ = 37% (18)
Nephtys hombergii
R^2 = 0.17 (0.23)
$RMSE_{bt}$ = 24% (18)
R^2 = 0.2 (0.24)
$RMSE_{bt}$ = 42% (28)
Scoloplos armiger
R^2 = 0.58 (0.23)
$RMSE_{bt}$ = 23% (10)
R^2 = 0.48 (0.28)
$RMSE_{bt}$ = 23% (10)
Marenzelleria viridis
R^2 = 0.69 (0.13)
$RMSE_{bt}$ = 74% (19)
R^2 = 0.61 (0.22)
$RMSE_{bt}$ = 109% (42)
abundance (n m^{-2})
biomass (g m^{-2})
occupancy (fraction of sampling stations)

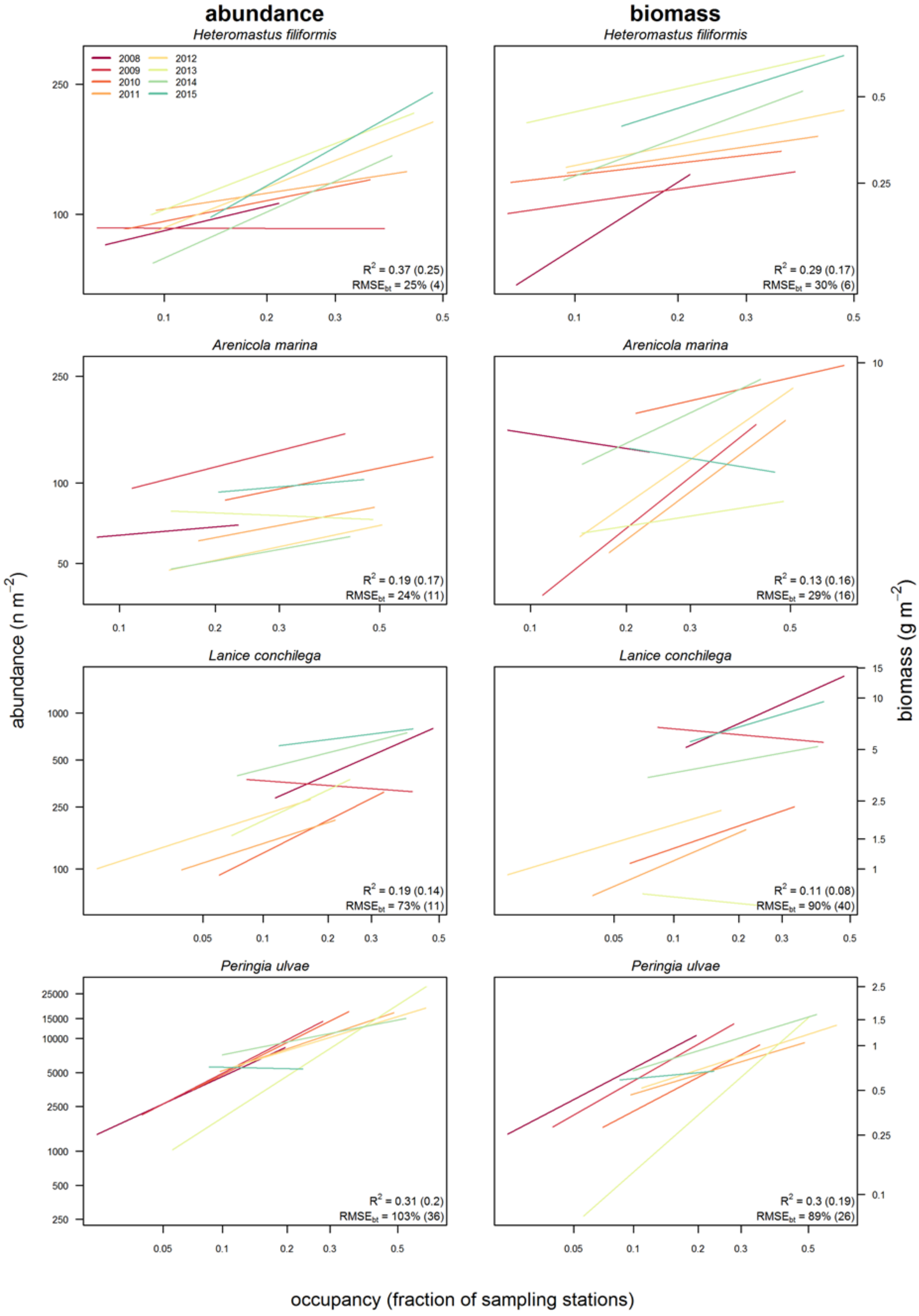
abundance
biomass
Heteromastus filiformis
2008
2009
2010
2011
2012
2013
2014
2015
R² = 0.37 (0.25)
RMSE_bt = 25% (4)
R² = 0.29 (0.17)
RMSE_bt = 30% (6)
Arenicola marina
R² = 0.19 (0.17)
RMSE_bt = 24% (11)
R² = 0.13 (0.16)
RMSE_bt = 29% (16)
Lanice conchilega
R² = 0.19 (0.14)
RMSE_bt = 73% (11)
R² = 0.11 (0.08)
RMSE_bt = 90% (40)
Peringia ulvae
R² = 0.31 (0.2)
RMSE_bt = 103% (36)
R² = 0.3 (0.19)
RMSE_bt = 89% (26)
abundance (n m⁻²)
biomass (g m⁻²)
occupancy (fraction of sampling stations)

Fig. S5 The difference in strength of intraspecific relationships according to the scale of the analyses. Abundance-occupancy relationships are shown in the left column, and biomass-occupancy relationships are shown in the right column. Each row represents a spatiotemporal comparison of relationships: comparing the temporal relationship within tidal basins and that of the entire Dutch Wadden Sea (small vs regional), comparing spatial relationships with temporal relationships for the entire Dutch Wadden Sea (spatial vs regional), and comparing spatial relationships with temporal relationships within tidal basins (spatial vs local). For each species and comparison, we calculated the difference (in percentage points) for the back-transformed Root Mean Squared Error ($RMSE_{bt}$) and coefficient of determination (R^2) by subtracting the statistics of the first mentioned scale from the second. For example, positive values in the first row of panels indicate that the local temporal relationship were stronger than the regional temporal relationships. For comparisons of temporal relationships within tidal basins, and spatial relationships between tidal basins, we used median values of R^2 and $RMSE_{bt}$. To guide the eye, we indicate the bivariate distribution of change (ellipse) with its centroid (cross), and indicate an increase in the strength of a relationship (shaded area). To indicate the absolute strength of relationships, we present filled symbols if the relationship has an $RMSE_{bt}$ below and R^2 above 50%. Different plotting symbols represent different taxonomic groups (see legend).

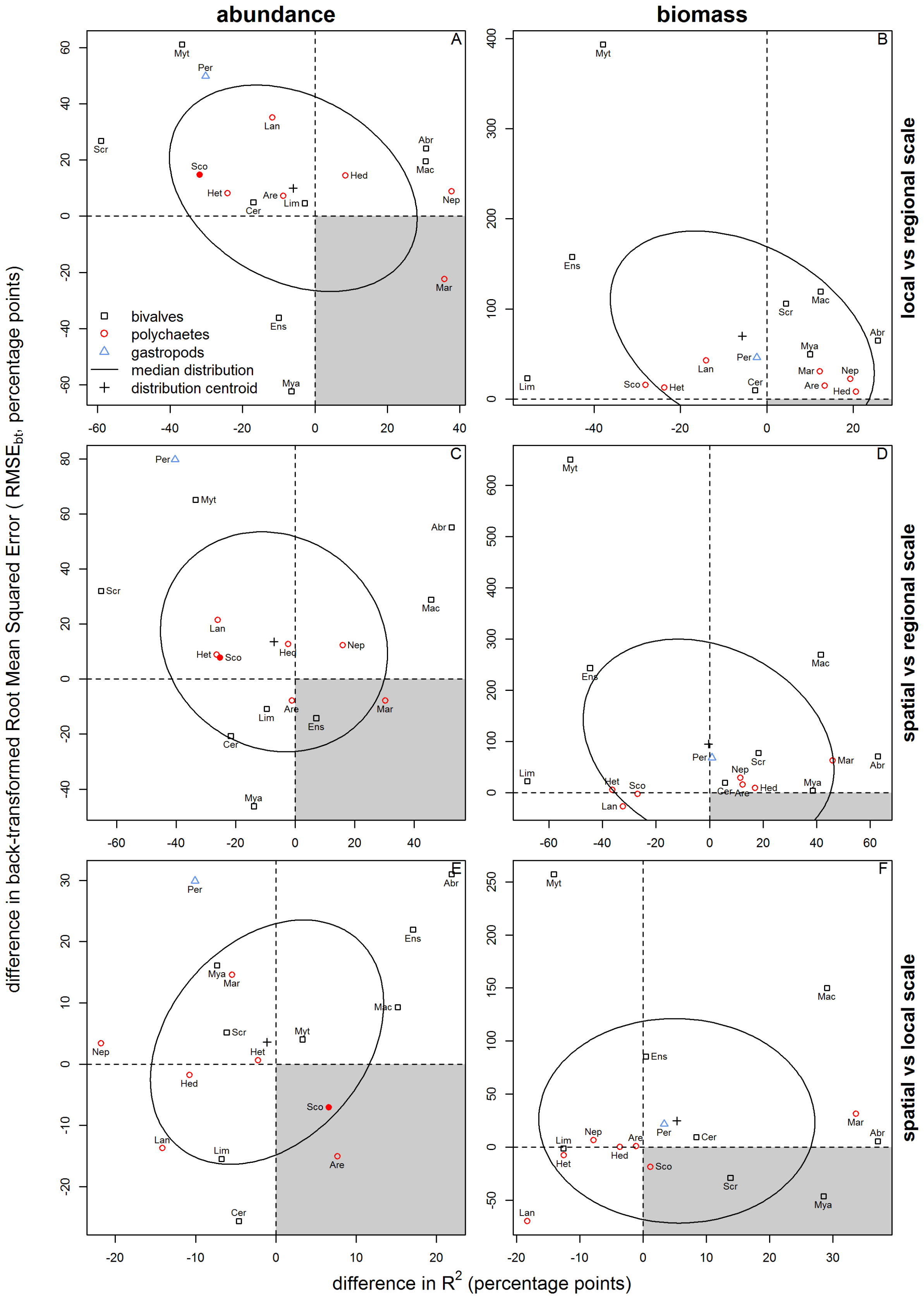

abundance
biomass
local vs regional scale
spatial vs regional scale
spatial vs local scale
A
B
C
D
E
F
bivalves
polychaetes
gastropods
median distribution
distribution centroid
difference in back-transformed Root Mean Squared Error (RMSE$_{bt}$, percentage points)
difference in R^2 (percentage points)

Appendix A1 – R script for identifying outliers

```
outliers <-function(afdm, L, criterium=2, span=span){
	# afdm = biomass of individuals
	# L = length of individuals
	# criterium = value to define outliers (the number of times the Inter quartile Range)
	# data frame needs to be ordered on the predictor variable for smoother
	d<-data.frame(ID=1:length(afdm), afdmi=afdm, L=L)

	## transform variables
	d$log_afdm<-log10(d$afdmi)
	d$log_L<-log10(d$L)

	## drop na's
	d2<-d[complete.cases(d),]
	d2<-d2[order(d2$L),]

	# fit non-linear smoother
	smoother <- loess(log_afdm ~ log_L, span=span, data=d2, control =
loess.control(surface = "direct"))

	#calculate residual flesh mass
	R<-d2$rel_afdm <- resid(smoother)

	## identify outliers
	#hist(R, n=30)
	resid.q <- quantile(R,prob=c(0.25,0.75))	#calaculate quantiles
	iqr <- diff(resid.q)	#calculate Inter Quartile Range
	limits <- resid.q + criterium*iqr*c(-1,1)
	#abline(v=limits, col=2, lty=2)
	score <- (pmin((R-limits[1])/iqr,0) + pmax((R - limits[2])/iqr,0))
	d2$outlier<-abs(score)>0

	## predict values on original dataframe
	d$predicted<-10^predict(smoother, log10(d$L))
	## match outliers
	id<-match(d$ID, d2$ID)
	d$outliers<-d2$outlier[id]

	# plot fit
	plot(smoother,xlab="",ylab="")
	title(ylab=expression(paste(log[10],"(",AFDM[flesh],", mg)")), outer=F, line=2.5,
cex.lab=1.25)
	title(xlab=expression(paste(log[10],"(length, mm)")), outer=F, line=3, cex.lab=1.25)

	lines(smoother$fitted~d2$log_L, col="green", lwd=3)
	points(d2$log_afdm[d2$outlier] ~ d2$log_L[d2$outlier], col=2, cex=2, pch=19)

	## plot outlier envelope
	L<-seq(min(d2$log_L),max(d2$log_L),0.01)
	AVG<- predict(smoother, L)
```

```
AVGmin<- AVG + limits[1]
AVGmax<- AVG + limits[2]
lines(AVGmin ~ L, col=2, lty=2)
lines(AVGmax ~ L, col=2, lty=2)

return(d)
}
```
